# A Delphi survey to determine how educational interventions for evidence-based practice should be reported: Stage 2 of the development of a reporting guideline

**DOI:** 10.1186/1472-6920-14-159

**Published:** 2014-07-31

**Authors:** Anna C Phillips, Lucy K Lewis, Maureen P McEvoy, James Galipeau, Paul Glasziou, Marilyn Hammick, David Moher, Julie K Tilson, Marie T Williams

**Affiliations:** 1 School of Health Sciences, University of South Australia, GPO box 2471, Adelaide 5001, Australia; 2 Health and Use of Time Group (HUT), Sansom Institute for Health Research, University of South Australia, GPO Box 2471, Adelaide 5001, Australia; 3 International Centre for Allied Health Evidence (iCAHE), School of Health Sciences, University of South Australia, GPO box 2471, Adelaide 5001, Australia; 4 Ottawa Hospital Research Institute, Centre for Practice-Changing Research (CPRC), The Ottawa Hospital, 501 Smyth Rd, Ottawa, Ontario K1H 8L6, Canada; 5 Centre for Research in Evidence-Based Practice (CREBP), Bond University, University Drive, Robina, Queensland 4226, Australia; 6 Bournemouth University, Royal London House, Christchurch Road, Bournemouth, Dorset, UK; 7 Ottawa Hospital Research Institute, Clinical Epidemiology Program, Centre for Practice-Changing Research (CPCR), The Ottawa Hospital, 501 Smyth Rd, Ottawa, Ontario K1H 8L6, Canada; 8 University of Southern California, Division of Biokinesiology and Physical Therapy, 1540 Alcazar St, CHP155, Los Angeles 90089, USA; 9 School of Population Health, Nutritional Physiology Research Centre (NPRC), University of South Australia, GPO Box 2471, Adelaide 5001, Australia

**Keywords:** Evidence-based practice, Reporting guideline, Delphi survey

## Abstract

**Background:**

Undertaking a Delphi exercise is recommended during the second stage in the development process for a reporting guideline. To continue the development for the Guideline for Reporting Evidence-based practice Educational interventions and Teaching (GREET) a Delphi survey was undertaken to determine the consensus opinion of researchers, journal editors and educators in evidence-based practice (EBP) regarding the information items that should be reported when describing an educational intervention for EBP.

**Methods:**

A four round online Delphi survey was conducted from October 2012 to March 2013. The Delphi panel comprised international researchers, educators and journal editors in EBP. Commencing with an open-ended question, participants were invited to volunteer information considered important when reporting educational interventions for EBP. Over three subsequent rounds participants were invited to rate the importance of each of the Delphi items using an 11 point Likert rating scale (low 0 to 4, moderate 5 to 6, high 7 to 8 and very high >8). Consensus agreement was set a priori as at least 80 per cent participant agreement. Consensus agreement was initially calculated within the four categories of importance (low to very high), prior to these four categories being merged into two (<7 and ≥7). Descriptive statistics for each item were computed including the mean Likert scores, standard deviation (SD), range and median participant scores. Mean absolute deviation from the median (MAD-M) was also calculated as a measure of participant disagreement.

**Results:**

Thirty-six experts agreed to participate and 27 (79%) participants completed all four rounds. A total of 76 information items were generated across the four survey rounds. Thirty-nine items (51%) were specific to describing the intervention (as opposed to other elements of study design) and consensus agreement was achieved for two of these items (5%). When the four rating categories were merged into two (<7 and ≥7), 18 intervention items achieved consensus agreement.

**Conclusion:**

This Delphi survey has identified 39 items for describing an educational intervention for EBP. These Delphi intervention items will provide the groundwork for the subsequent consensus discussion to determine the final inclusion of items in the GREET, the first reporting guideline for educational interventions in EBP.

## Background

The philosophy of evidence-based practice (EBP) dates back to the late 1600’s [1]. Although the definition of EBP has evolved over the last four centuries, the primary purpose remains unchanged [2-4]. Evidence-based practice provides a framework for health professionals to guide clinical decision-making to produce optimal patient care [2-5].

The importance of education in EBP is widely recognised, and there has been an almost universal uptake of teaching EBP in health professional programs [6]. The number of research reports of educational interventions in EBP has risen considerably, with over 170 studies reporting an EBP educational intervention identified in a recent systematic review [7]. Despite this investment of time, effort and expertise from researchers and educators in EBP education, best practice for the teaching of EBP remains unknown [8].

Further development of the evidence-base for education in EBP is needed, however educational interventions are complex, as are the systems in which they are conducted and these complexities pose significant challenges in the design, evaluation and reporting of educational interventions [9]. In two recent systematic reviews of EBP educational interventions, Ilic & Maloney [8] and Maggio [10] highlighted the need for improvements in the level of detail provided in the reporting of the description of the intervention to enable conclusions to be drawn regarding the efficacy of educational interventions for EBP. Reporting guidelines, with the intent of enabling consistent and transparent reporting of studies have existed for almost two decades [11] and there are over 200 reporting guidelines listed on the EQUATOR network [12]. Many of these reporting guidelines are for specific study designs such as CONSORT for randomised controlled trials [13], STROBE for observational studies [14] and TREND for non-randomised trials [15]. Few reporting guidelines exist for the reporting of interventions [16] with only four reporting guidelines currently available for describing specific educational interventions [17-20]. To date, there are no specific reporting guidelines for reporting educational interventions used to develop knowledge and skills in EBP.

To enable the standardised and transparent reporting of educational interventions for EBP, we have proposed the development of a reporting guideline, the guideline for reporting evidence-based educational interventions and teaching (GREET) [21]. Rather than replicate reporting guidelines for study designs, the intent of the GREET is that it should provide detailed information for describing the intervention only and should be used in conjunction with existing reporting guidelines for study design. Comprising a three stage process, the development for the GREET included a systematic review of the literature concerning EBP educational interventions for health professionals in stage 1 [21]. Reviewing randomised and non-randomised studies that investigated an educational strategy for facilitating knowledge and skills of EBP resulted in a list of items which have been commonly reported when describing educational interventions.

The second stage in the development process for GREET was to undertake a Delphi survey to elicit a prospective expert consensus opinion regarding the information items that should be included in the reporting guideline [22]. The Delphi method is an anonymous iterative process that comprises a series of rounds of questionnaire, response and feedback until consensus is achieved [23].

The aim of this study was to determine the consensus opinion of researchers, educators and journal editors regarding which items should be reported when describing an EBP educational intervention using a Delphi process [24,25].

## Methods

Ethical approval was obtained from the University of South Australia Human Research Ethics Committee (protocol no. 25590).

### Participants

Invitations for the Delphi survey were sent to corresponding authors of the 61 studies included in a recent systematic review [26] and to the editors of the 34 journals in which these studies were published. A return email accepting the invitation constituted participants’ consent to participate.

#### ***Procedure***

The Delphi process was commenced in October 2012 and completed in March 2013. A maximum of four survey rounds were prospectively planned or until consensus agreement was reached. Feedback of the results from the previous round was provided in each subsequent round [25]. All participants were invited to complete each and every Delphi round, regardless of participation in the previous round, unless they indicated withdrawal from the study.

Reminders were sent at seven and 14 days following the dissemination of each survey round. The Delphi survey round closed 21 days after the initial survey was sent.

##### 

**Data collection** In order to enable efficient and timely data collection from an international pool of participants, an electronic survey instrument was used (SurveyMonkey^®^).

##### 

**Development of Round 1 survey** The aim of the first round survey was to generate a list of items participants considered should be reported when describing an EBP educational intervention. An open ended question, rather than a pre-determined list of items was used to minimise potential publication or personal biases [25]. The initial draft survey was pilot tested by four staff members with expertise in EBP research and the practice and teaching of EBP from the International Centre for Allied Health Evidence (iCAHE) at the University of South Australia, who were not involved in the Delphi [25]. Pilot testing determined the accessibility of the electronic survey, the time taken to complete the survey, clarity of the wording, layout and ease of use. After minor amendments arising from the pilot testing, the Round 1 survey format was considered complete.

The initial survey comprised three sections: a brief overview of the Delphi process, demographic information, and one open ended question asking participants which items should be included when describing an educational intervention for EBP. An example from a study which provided limited detail in the reporting of the educational intervention for EBP was provided as a prompt for participants to help identify information relevant for the reporting of the intervention. Space was provided for further comments from participants.

Example of the open ended question from the Round 1 Delphi survey:

The following paragraph presents an example of the information provided in a published study. *“Participants in the intervention group received an evidence based practice course of three half days spread over 2 weeks. During this course they learned the basics of evidence based practice. Upon completion of the evidence- based practice course participants scheduled 10 learning sessions with their peer group. These sessions took place every other week and lasted 1–1.5 hours”*[23].

If you were reading a study which reported an educational process for facilitating foundation skills in evidence- based practice (ask, acquire, appraise, apply and assess) what information about the INTERVENTION would you expect to be included?

#### ***Round 1 survey***

Round 1 (including an electronic link to the online survey) was sent via email. Participants were reminded of the importance of completing all four rounds to minimise attrition bias and that participation was voluntary.

##### 

**Data management** Each participant was allocated a random identification number for reporting and collation of the results. Demographic data were collated and summarised for the group. All responses to the open ended question were downloaded verbatim to a spread sheet (*Excel.* Version 14. Microsoft; 2010). All information items volunteered by participants were reviewed and allocated to one of five domains (Table 1).

**Table 1:** The five domains used for allocation of the volunteered Delphi items

**Domain**	**Information extracted**
1 Participants	Learners and instructors in intervention
2 Intervention	Educational/theoretical framework for the intervention
	How the intervention was delivered (e.g. number of sessions, duration of sessions)
	Setting where the intervention was undertaken
3 Content	Learning objectives for intervention
	Content of EBP included in intervention
4 Evaluation	All methods of assessment used for the learners
5 Other	All information that did not fit into the previous four domains such as information regarding study design, methodology and study limitations.

The Principal Investigator (AP) allocated each response using pre-determined coding [21]. The allocation of items was independently reviewed by at least one other member of the research team (MTW, MPM, LKL). Where there was uncertainty about the coding for an item, the coding was discussed with the research team until consensus agreement was reached.

#### ***Round 2–4 surveys***

Rounds 2, 3 and 4 aimed to fulfil the consensus process.

In each round, participants were provided with a summary of the results from the previous round [27], instructions for completing the survey and the electronic survey link.

Participants were invited to rate the importance of each item on an 11-point Likert scale ranging from zero (no importance = not essential for reporting) to 10 (highest importance = essential for reporting). In addition, participants were invited to provide a brief justification or cite a relevant study to support their rating scores. The final section of the survey sought to elicit whether respondents found any of the items unclear, wanted to suggest any further items or add any other comments. At the end of the second round, the information items volunteered by the Delphi participants were cross checked against the items reported in the systematic review [21] to ensure that all information items in the systematic review were included for review and rating in the Delphi survey [21].

##### 

**Consensus** Consensus was determined *a priori*[25]. For an item to achieve consensus, 80 per cent or more of respondents must have rated the item in the same category of importance using an 11 point Likert scale (low importance 0 to 4, moderate importance 5 to 6, high importance 7 to 8 or very high importance >8).

##### 

**Feedback** Based on the Delphi recommendations by the RAND Corporation [27], a participant feedback report which summarised the findings from the previous round was compiled for Rounds 2, 3 and 4. The feedback document was provided to participants one week prior to the commencement of the following round, or one month after the completion of the final round.

##### 

**Data management** The total number of completed surveys (number of participants) and the Likert rating score for each item for each respondent was recorded. Descriptive statistics for each item were computed, including the mean Likert scores, standard deviation (SD), range and median participant scores. Mean absolute deviation from the median (MAD-M) was also calculated as a measure of participant disagreement [27]. Likert scores for each item, per participant, were allocated into categories of importance (low: 0 to 4; moderate: 5 to 6; high: 7 to 8 and very high: > 8) and per cent agreement (frequency of respondents) was calculated for each category.

In the final round, where specific items did not reach the pre-determined level of consensus (>80% agreement), items were assigned categories of importance based on the greatest participant agreement within these four categories. Items with the greatest participant agreement in the low importance category (Likert scores 0 to 4) were deemed *unlikely* to be included in the GREET; items with the greatest participant agreement in the moderate importance category (Likert scores 5 to 6) were characterised as *could be considered* for inclusion in the GREET and items with the greatest participant agreement in the high to very high importance category (Likert scores ≥7), were characterised as *likely to be included* in the GREET.

## Results

### Participant characteristics

The uptake rate for the Delphi survey was 34 per cent, with 36 out of the 105 potential participants accepting the invitation to participate (Table 2). Two participants withdrew over the course of the Delphi survey (one after Round 1 and Round 3), resulting in 34 participants for Round 4. Response rates across the four rounds were 100% (R1), 94% (R2), 97% (R3) and 97% in Round 4. A total of 27 out of the final 34 participants responded to all four rounds, achieving an overall response rate of 79 per cent.

**Table 2:** Participant’s characteristics and responses

**Participants**	**Authors (n = 28) n (%)**	**Editors (n = 8) n (%)**
*Round (n)*		
1 (n = 36)	28 (78)	8 (22)
2 (n = 35, 1 withdrew)	26 (74)	7 (20)
3 (n = 35)	26 (74)	8 (23)
4 (n = 34, 1 withdrew)	22 (65)	5 (14)
*Sex*		
Female	15 (54)	5 (62)
Male	13 (46)	3 (38)
*Professional role*		
Research and teaching	15 (54)	4 (50)
Teaching	4 (14)	
Clinician	4 (14)	
Researcher in EBP education	3 (11)	2 (25)
Other*	2 (7)	2 (25)
*Professional discipline*		
Medicine	12 (43)	2 (25)
Nursing	4 (14)	1 (13)
Librarian	3 (11)	2 (25)
Social work/social science	2 (7)	1 (12)
Other*	7 (25)	2 (25)
*Highest qualification*		
Doctorate	22 (79)	6 (75)
Medical Doctor	3 (11)	
Masters	2 (7)	2 (25)
Honours	1 (3)	
*Experience*		
>10 years	27 (96)	7 (88)
5-10 years	1 (4)	
<2 years		1 (12)
*Country*		
United states	11 (39)	4 (50)
United Kingdom	5 (18)	2 (24)
Canada	5 (18)	1 (13)
Hong Kong	2 (7)	
Australia	1 (4)	1 (13)
Netherlands, New Zealand, Norway, Switzerland (1 each)	4 (14)	

*other Professional roles = Public Health director (n = 2), Journal Editor (n = 2) and all of the above (n = 2).

*other Professional disciplines Clinical or Cognitive Psychologist, Public Health (n = 2), Academic Development, Epidemiologist, Information Science, Health Informatics, Statistician (n = 1).

### Item generation

A total of 344 items were volunteered by participants in Round 1, with an average of 10 items per participant (range 0–24 items). After the removal of duplicate items (n = 276), 68 items were categorised into the five pre-determined domains. Eight additional items were added after Round 2 (six items added after cross checking items derived from the systematic review and two additional items that were volunteered by participants). No further items were added after Rounds 3 and 4. There were 76 items generated in total from the Delphi process.

### Delphi items specific to describing the intervention

As the intent of the Delphi survey was to determine which information items participants considered important for describing an intervention in EBP education, further review of the 76 items was undertaken. This was done to identify items that related to study design or methodology (and therefore not specific to this study aim), from items that related to the description of the *intervention*. When the 76 Delphi items were reviewed using reporting guidelines specific for research design (CONSORT) [13] and generic interventions [Template for Intervention Description and Replication (TIDieR)] [16], 39 items (51%) were identified as descriptors of the intervention (Table 3). There were therefore 37 items that were considered not to be related to the *intervention*.

**Table 3:** Summary of round 4 ratings for Delphi intervention items (n = 39)

**Information item**	**n**	**Mean (SD)**	**Median**	**MAD-M**	**Frequency (%) per category of importance**				**Include in GREET**
					**V high**	**High**	**Mod**	**Low**	
Aims and objectives of the educational intervention*	26	10.0 (0.9)	10.0	0.6	85	15	0	0	Yes
Teaching/learning strategies^+^	26	9.5 (1.6)	10.0	1.1	69	23	4	4	Yes
Learning objectives*	26	9.4 (1.1)	10.0	0.6	81	19	0	0	Yes
Duration of each session^+^	26	9.4 (1.5)	9.0	1.1	69	23	8	0	Yes
Number of face to face teaching/learning sessions^+^	26	9.3 (1.7)	9.0	1.2	69	23	4	4	Yes
Duration of each entire educational program^+^	26	9.3 (2.0)	9.0	1.1	73	19	4	4	Yes
Frequency of the teaching/learning sessions^+^	26	9.3 (2.8)	9.0	1.1	73	19	4	4	Yes
Any post-intervention activities required^+^	26	9.2 (1.3)	9.0	1.0	62	30	8	0	Yes
Theoretical basis/educational framework used^+^	26	9.0 (1.6)	9.0	1.3	50	30	20	0	Yes
The specific educational materials/resources used^+^	26	9.0 (1.9)	9.0	1.3	50	38	12	0	Yes
Any pre-intervention readings/activities required^+^	26	8.9 (1.3)	8.5	1.1	50	46	4	0	Yes
Detail of EBP components/content^+^	25	8.9 (1.4)	9.0	1.0	68	28	4	0	Yes
Process used to ensure fidelity of teaching/delivery	25	8.9 (1.8)	9.0	1.1	56	16	28	0	Likely
Timing of intervention	26	8.0 (2.3)	8.0	1.6	30	46	12	12	Likely
Supporting structures in organisation to maintain behaviours targeted by intervention^+^	26	7.9 (1.4)	8.0	1.0	34	54	12	0	Yes
Extent of peer interaction	25	7.9 (2.3)	8.0	1.5	24	52	12	12	Likely
What post-training support was provided^+^	26	7.8 (1.7)	8.0	1.4	38	42	15	5	Yes
Face to face contact time with learners^+^	26	7.8 (1.9)	8.0	1.3	38	46	8	8	Yes
Whether any identified barriers were targeted ^+^	26	7.6 (1.4)	7.5	1.2	27	58	15	0	Yes
Whether follow-up sessions planned^+^	26	7.5 (1.7)	8.0	1.2	27	54	15	4	Yes
Training required for instructors to teach the intervention	25	7.3 (1.7)	7.0	1.4	28	40	32	0	Likely
Non-face to face contact time with learners	26	7.2 (2.1)	8.0	1.6	27	42	19	12	Likely
Instructors commitment to specific content of teaching	26	7.2 (2.1)	8.0	1.5	27	42	23	8	Likely
Student time NOT covered by face to face contact	26	7.2 (1.8)	8.0	1.4	19	50	23	8	Likely
What method was used to decide content	25	7.1 (2.3)	7.0	1.9	32	32	20	16	Likely
Number of instructors/teachers involved	25	7.0 (1.8)	7.0	1.2	12	60	24	4	Likely
Ratio of learners to teachers	25	6.9 (1.8)	7.0	1.1	12	64	16	8	Likely
Instructors commitment to format of teaching	26	6.8 (2.3)	8.0	1.7	20	42	23	15	Likely
Whether the same instructor was used for all teaching	25	6.8 (2.0)	7.0	1.4	16	52	24	8	Likely
Whether a systematic method was used beforehand to identify barriers	26	6.8 (1.5)	6.0	1.2	15	31	50	4	Consider
Whether program will be compared across different sites	26	6.6 (2.4)	7.0	1.8	20	42	23	15	Likely
Settings where teaching/learning sessions undertaken	26	6.5 (1.9)	7.0	1.2	8	62	15	15	Likely
Description of teaching experience/expertise	24	6.5 (1.6)	6.0	1.2	13	38	42	12	Consider
Profession of instructors	25	6.1 (2.6)	7.0	2.0	12	40	24	24	Likely
Whether educational intervention was endorsed by an academic, educational or professional institution	27	6.1 (2.7)	7.0	2.1	22	26	33	19	Consider
Who was involved in designing the content	26	5.7 (2.8)	6.0	2.3	23	15	35	27	Consider
Relation of instructor to learners/program	26	5.5 (2.2)	5.0	1.5	12	12	50	26	Consider
Who designed the intervention	26	5.2 (3.3)	5.0	2.5	27	8	19	46	Unlikely
To what extent did the hosting agency facilitate training^+^	26	4.8 (2.1)	5.0	1.5	8	12	46	34	No

*item achieved consensus agreement (≥80%) using original four categories of agreement, ^+^item achieved consensus agreement (≥80%) using collapsed categories of agreement.

The Round 4 ratings of importance for these 39 intervention items across categories of importance were; very high (n = 10, 26%), high (n = 16, 41%), moderate (n = 12, 31%) and low (n = 1, 3%) (Table 3). The intervention items achieving the highest ratings by participants were A*ims and objectives of the educational intervention, Teaching/learning strategies* and *Learning objectives,* all achieving a median Round 4 rating score of 10. The intervention items achieving the lowest participant rating scores were *To what extent did the hosting agency facilitate the training, Who designed the intervention* and *The relation of the instructor to the learner/program,* all achieving a median Round 4 rating score of 5.

### Consensus

Consensus agreement was determined *a priori* as greater than 80 per cent participant agreement within one of the four categories of importance. Two intervention items, *Aims and objectives of the educational intervention* [mean rating 10.0 (0.9), median 10.0, MAD-M 0.6] and *Learning objectives* [mean rating 9.4 (1.1), median 10.0, MAD-M 0.6] achieved consensus agreement (Table 3). When the four categories of importance were merged into the two categories of low to moderate importance (<7) and high to very high importance (≥7), a further 16 items achieved consensus agreement (Table 3). With the exception of the item *To what extent did the agency hosting it facilitate the training* [mean score 4.8(2.1), median 5.0, MAD-M 1.5], all items (n = 17) were rated as of high to very high importance for reporting (Table 3).

### Items not reaching consensus

The remaining 21 items (54%) that did not achieve consensus agreement using either the *a priori* criterion or the merged categories were classified according to the category with the greatest participant agreement. The majority of items (n = 15, 71%) had the greatest participant agreement in the high to very high importance category (Likert scores ≥7), reflecting that these items were likely to be included in the GREET (Table 3). Five items, *Whether a systematic method was used beforehand to identify barriers*, *Who was involved in designing the content, Relation of the instructor to learners/program, Whether the educational intervention was endorsed by an academic, educational or professional institution and Description of teaching experience/expertise*, were classified as could be considered for inclusion in the GREET, with greatest participant agreement in the moderate importance category (Likert scores 5 to 6). One item, *Who designed the intervention*, was considered unlikely to be included in the GREET, with the greatest participant agreement in the low importance category (Likert scores <5) (Table 3).

### Participant justifications and comments

Although participants were invited to provide a brief justification or to cite a relevant study to support their rating scores, no citations were provided during any round of the Delphi.

Several participants provided comments for the Delphi items, with a total of 111 comments for the intervention items across Rounds 2–4. The greatest number of participant comments was provided in Round 2 [mean 2(1), median 2]. There was no apparent relationship between the number of participant comments, the rating of importance or the level of participant agreement for the items. For descriptive purposes, the participants’ comments were allocated into four categories (Table 4). The four categories used to describe the comments were as follows:

**Table 4:** Summary of number and type of comments provided by Delphi participants for intervention items

**Information item**	**Number of participants**	**Reinforce n = 76 (68%)**	**Replicate n = 14 (13%)**	**Understand n = 14 (13%)**	**Philosophy n = 7 (6%)**
Aims and objectives of the educational intervention	3	x	xx		
Teaching/learning strategies	5	xxxx	x		
Learning objectives	7	xxxxx	xx		
Duration of each session	2	xx			
Number of face to face teaching/learning sessions	7	xxxx	xx		x
Duration of each entire educational program	1	x			
Frequency of the teaching/learning sessions	0				
Any post-intervention activities required	1	x			
Theoretical basis/educational framework used	5	xxxx	x		
The specific educational materials/resources used	3	xx	x		
Any pre-intervention readings/activities required	0				
Detail of EBP components/content	2	x	x		
Process used to ensure fidelity of teaching/delivery	7	xx		xxx	xx
Timing of intervention	3	xx		x	
Supporting structures in organisation to maintain behaviours targeted by intervention	3	xxx			
Extent of peer interaction	0				
What post-training support was provided	0				
Face to face contact time with learners	8	xxxxxx	x		x
Whether any identified barriers were targeted	2	x			x
Whether follow-up sessions planned	1	X			
Training required for instructors to teach the intervention	1	x			
Non-face to face contact time with learners	2	xx			
Instructors commitment to specific content of teaching	5	xx		xx	x
Student time NOT covered by face to face contact	2	xx			
What method was used to decide content	1			x	
Number of instructors/teachers involved	4	xx	x	x	
Ratio of learners to teachers	5	xxx	x	x	
Instructors commitment to format of teaching	5	xxxx		x	
Whether the same instructor was used for all teaching	3	xx			x
Whether a systematic method was used beforehand to identify barriers	1			x	
Whether program will be compared across different sites	3	xxx			
Settings where teaching/learning sessions undertaken	4	xx		xx	
Description of teaching experience/expertise	3	xx	x		
Profession of instructors	4	xxxx			
Whether educational intervention was endorsed by an academic, educational or professional institution	2	xx			
Who was involved in designing the content	1	x			
Relation of instructor to learners/program	4	xxx		x	
Who designed the intervention	1	x			
To what extent did the hosting agency facilitate training	0				

(1) Reinforcing the participant’s rating assigned for the item

Most of the comments provided by participants (n = 76, 68%) were related to reinforcing or justifying their rating of importance for the information items. An example of a comment provided by a participant, “*Obviously the teaching strategies and objectives are essential for readers to understand the intervention and also in determining if the objectives are met (by student achievement and by the intervention teaching strategy).”*

(2) Enabling replication of the intervention

This was the second most frequent category for the participants’ comments (n = 14, 13%), with comments relating to the importance for the information item to enable replication of the intervention.

An example of a comment provided by a participant, “*Almost all of these are essential in giving enough detail so that the study could be reproduced.”*

(3) Clarification of an item

There were 14 comments (13%) relating to seven different items which participants stated were either unclear or they did not understand the meaning of the information item. For example, *“I'm unclear what the intended meaning of the word 'commitment' is within the context of this question. ..?”*

(4) Philosophical perspectives concerning an item

Participants expressed a philosophical opinion regarding the item. This was least common category for the participants’ comments with seven (6%) comments relating to a philosophical or pedagogical perspective for the information item.

An example of a comment provided by a participant *“I rankled at having to respond to this because it makes it seem as though there is only one right way to teach something. I think that one of the reasons that teaching is such a complex skill is because a good teacher can recognise when a different way is needed and they are able to modulate the way they teach to meet the learning needs of the students. However, I realised that, for some, anxiety arises if they are not taught what they considered to be the 'intended content' and the 'intended delivery method”* (Table 4).

## Discussion and conclusions

The purpose of a Delphi survey is to use an iterative process to combine expert opinion into group consensus [28]. Consensus agreement does not mean that the correct answer has been found, but rather that a level of participant agreement has been reached [25]. The information items resulting from this Delphi survey represent the opinion of an expert panel regarding which information should be reported when describing an EBP educational intervention.

The electronic survey process proved to be a successful, feasible and cost efficient method. Four rounds of Delphi survey and response were completed over a six month period and 79 per cent of participants completed all four Delphi rounds. A total of 76 items were assessed during this Delphi process, with 39 items (51%) relating specifically to the description of the educational intervention. Almost half of the intervention items (n = 18, 46%) achieved consensus agreement in the two merged categories of importance (<7 and ≥7).

Attempts were made to invite a representative panel which included stakeholders in EBP education, research and editorial responsibilities. The final Delphi panel comprised 36 participants, which was larger than the average number of 24 participants involved previous Delphi surveys for reporting guideline development [29] and within the range of 10 to 1685 participants used in previous Delphi surveys [30].

Many of the Delphi intervention items do not seem unexpected for consideration or inclusion in the reporting guideline. Without adequate description of information such as the aims and objectives, learning objectives, number, duration, frequency of the learning sessions, theoretical basis/educational framework, educational materials/resources used, EBP content for the intervention, it is not possible to implement the educational intervention or enable adaption in other settings [9]. Many of the items achieving consensus agreement, including the teaching/learning strategies, educational methods (e.g. lecture, case based discussion), educational/theoretical framework and setting for the educational intervention have been previously included as suggestions for reporting educational interventions [9].

### Limitations

There are several potential limitations identified for this study. Firstly, despite the intent to invite a Delphi panel that was representative of authors who had completed an educational intervention study for knowledge and skills in EBP, and journal editors from the journals in which these studies were published, the final Delphi panel was comprised of a predominance of authors (n = 28, 78%) who were medical professionals (n = 14, 39%), nurses and librarians (n = 5, 17%). Most participants were North American (n = 22, 61%), and there were no Delphi participants from developing countries. It should be noted that studies from developing countries were under represented in the systematic review undertaken in stage 1 of the development for GREET, with only one study (2%) from developing countries (Mexico – Sanchez-Mendiola 2004) [31]. The corresponding author of this study was invited to participate in the Delphi survey, but did not accept our invitation. It is unclear how input from authors and journal editors from the developing world may have impacted on the results of this Delphi survey.

Secondly, there are no current recommendations for determining the threshold for consensus agreement. In the absence of a gold standard method for determining consensus, the *a priori* level of agreement used in this study was based on previous Delphi surveys undertaken in the development for reporting guidelines and the recommendations from the RAND Corporation [27]. Despite excellent agreement for many items (evidenced by low MAD-M scores), a priori consensus agreement was only achieved for two items. This stringent level required for consensus may account for the small number of items achieving consensus agreement after four rounds. Furthermore, the allocation of four categories of importance resulted in narrow groupings for the Likert ratings. With the exception of low importance, each category spanned only two Likert ratings (low 0 to 4, moderate 5 to 6, high 7 to 8, very high 9 to 10). Merging the importance categories into two (<7 and ≥7) resulted in an eight-fold increase in the number of intervention items achieving consensus agreement (from two items to 18 items). On reflection, a 9 point Likert rating scale as recommended in the recently released RAND online resource [32], with three, three point rating categories may have been more appropriate.

There is an alternative scientific method to address agreement which is based on probabilities (p values) using the Wilcoxon signed rank test and interquartile range (IQR) [33]. Based on the outputs from Wilcoxon signed rank analyses, the recommendations for the inclusion of items in the GREET are very similar to the analysis we employed. The final recommendations for three items (*Relation of instructor to learner/program, Who was involved in designing the content and Whether the educational intervention was endorsed by an academic, educational or professional institution)* would change from consider for inclusion in the GREET, to unlikely to be included in the GREET. The outcome of the Delphi survey would have been similar, regardless of whether the current or alternative method was applied to determine consensus.

Thirdly, although every attempt to provide clear instructions and clarify the intent of the study, one conceptual issue arose throughout each Delphi round. For many of the participants, there was a lack of separation between items which might be included when reporting studies which include an educational intervention in EBP (for example, design, participants, methodology), versus items that are specific to the educational *intervention* itself. Almost half of the Delphi items (n = 37, 49%) did not relate to describing the intervention, which was the primary question posed by the Delphi survey. Furthermore, there was considerable overlap between many of the information items volunteered by the Delphi participants. However, in keeping with the intent of the Delphi process, irrespective of their interpretation, no items were discarded or modified by the researchers. All items were provided for participants to rate in terms of importance when describing the educational intervention.

Finally, despite the recommendation for Delphi surveys to undertake four rounds [25], no previous reporting guideline developers have used a four round Delphi process [29]. The advantages of four rounds of survey and responses include the opportunity for participants to rate the list of items on at least two occasions and to receive feedback over three consecutive rounds. A disadvantage associated with four rounds is greater participant burden and the possibility of participant fatigue, which may have been a factor in the slightly reduced response rate from 97 per cent in Round 3 to 79 per cent in the final round.

To assess the impact of the seven non-responders in Round 4, we compared demographic data and the Round 3 results with and without the non-responders’ ratings. Demographic data for the seven non-responding participants reflected the characteristics of participants who responded in all four rounds. When the responses of the seven Round 4 non-responders were excluded from the Round 3 analyses, two items would have achieved consensus in Round 3, rather than Round 4, suggesting that the non-responders had negligible impact on the overall results.

#### Implications for practice and future research

This Delphi survey completes the second stage in the development process for the Guideline for Reporting Evidence-based practice Educational interventions and Teaching (GREET). The systematic review undertaken in stage 1 of the development for the GREET, prior to this Delphi survey, has determined what has been previously reported in educational interventions for EBP. This Delphi survey, following on from the systematic review, has determined a consensus opinion regarding what information should be reported for educational interventions for EBP.

The findings of this Delphi survey propose a preliminary list of 39 intervention items, for further consideration within the GREET.

The next stage of the development process for the GREET will comprise a consensus meeting, which is intended to be conducted via international teleconference, to determine which of the intervention items will be included in the GREET. The development plan for the explanation and elaboration paper (E&E) to accompany the GREET, the pilot testing procedure to be undertaken and the publication and dissemination plan for the reporting guideline will also be determined during this discussion.

The standard of reporting for educational interventions for EBP remains inconsistent [8-10]. This means the most effective intervention for increasing EBP competency is not able to be determined despite the extensive investment of time and resources spent on educational interventions for EBP [8]. Olson and Bakken [9] list poorly described interventions as “*a common complaint of investigators undertaking systematic reviews on the effectiveness of educational interventions*”. Rather than accepting the status quo of inconsistent reporting, we are taking the first steps to address this issue and to enable the consistent and detailed reporting for educational interventions in EBP. The GREET will be the product of an explicit development process which aims to improve the transparency and consistency for reporting of educational interventions for EBP.

## Competing interests

Dr Moher is supported by a University Research Chair. Dr Moher is a member of the EQUATOR executive committee.

## Authors’ contributions

AP planned and carried out the Delphi survey, completed the analyses and drafting of the manuscript. LKL contributed to the planning stages for the Delphi survey, participated in the entire Delphi process including the analyses and writing of the manuscript. MPM participated in the planning and development for the entire Delphi process, including reviewing of the results, assisting with the analyses and drafting of the manuscript. JG and DM made substantial contributions to the analysis of the Delphi survey, particularly with respect to the determination of consensus. PG provided considerable input into the analysis of the Delphi survey and the determination of the Delphi intervention items. MH participated in the planning phase for the Delphi survey, assisted in reviewing the results from each round and writing of the manuscript. JKT contributed extensively to the drafting and critical revision of the manuscript. MTW contributed the original concept for the Delphi process and contributed to the undertaking and analysis of the Delphi process and helped write the manuscript. All authors read and approved the final manuscript.

## Authors’ information

AP is a PhD candidate, School of Health Sciences, University of South Australia, Adelaide, Australia.

LKL is a Post-doctoral Research Fellow, Health and Use of Time Group (HUT), Sansom Institute for Health Research, School of Health Sciences, University of South Australia, Adelaide, Australia.

MPM is a Lecturer, School of Health Sciences and a member of the International Centre for Allied Health Evidence (iCAHE), University of South Australia, Adelaide, Australia.

JG is a Senior Research Associate, Ottawa Hospital Research Institute, The Ottawa Hospital, Centre for Practice-Changing Research (CPCR), Ontario, Canada.

PG is the Director, Centre for Research in Evidence-Based Practice (CREBP), Bond University, Queensland, Australia.

DM is a Senior Scientist, Clinical Epidemiology Program, Ottawa Hospital Research Institute, The Ottawa Hospital, Centre for Practice-Changing Research (CPCR), Ontario, Canada.

JKT is an Associate Professor, University of Southern California Division of Biokinesiology and Physical Therapy, Los Angeles, USA.

MH is a visiting Professor, Bournemouth University, Bournemouth, UK and a consultant to Best Evidence Medical Education (BEME).

MTW is an Associate Professor, School of Population Health and a member of the Nutritional Physiology Research Centre (NPRC), School of Health Sciences, University of South Australia, Adelaide, Australia.

## Pre-publication history

The pre-publication history for this paper can be accessed here:

http://www.biomedcentral.com/1472-6920/14/159/prepub
